# Study on the Effects of Irrigation with Reclaimed Water on the Content and Distribution of Heavy Metals in Soil

**DOI:** 10.3390/ijerph13030298

**Published:** 2016-03-08

**Authors:** Shibao Lu, Jianhua Wang, Liang Pei

**Affiliations:** 1 School of Urban-rural Planning and Management, Zhejiang University of Finance and Economics, Hangzhou 310018, China; lu5111284@aliyun.com; 2 State Key Laboratory of Simulation and Regulation of Water Cycle in River Basin, China Institute of Water Resources and Hydropower Research, Beijing 100101, China; 3 Key Laboratory of Water Cycle and Related Land Surface Processes, Institute of Geographic Sciences and Natural Resources Research, Chinese Academy of Sciences, Beijing 100101, China; peiliang@igsnrr.ac.cn

**Keywords:** reclaimed water for irrigation, tomatoes, heavy metal pollution, soil-crops system, migration and transformation

## Abstract

Reclaimed water is an important resource for irrigation, and exploration in making full use of it is an important way to alleviate water shortage. This paper analyzes the effects of irrigation with reclaimed water through field trials on the content and distribution of heavy metals in both tomatoes and the soil. By exploring the effects of reclaimed water after secondary treatment on the content and distribution characteristics of heavy metals in tomatoes and the heavy metal balance in the soil-crop system under different conditions, the study shows that there are no significant differences in the heavy metal content when the quantity of reclaimed water for irrigation varies. Reclaimed water for short-term irrigation does not cause pollution to either the soil environment or the crops. Nor will it cause the accumulation of heavy metals, and the index for the heavy metal content is far below the critical value of the national standard, which indicates that the vegetables irrigated with reclaimed water during their growth turn out to be free of pollutants. The heavy metals brought into the soil by reclaimed water are less than that taken away by the crops. The input and output quantities have only small effects on the heavy metal balance in the soil. This paper provides a reference for the evaluation and safety control of irrigation with reclaimed water.

## 1. Introduction

Reclaimed water is a complementary water resource, which does not only reduce the amount of sewage, but also reduces the demand for high quality water [1,2]. The agricultural use of reclaimed water means that reclaimed water of different qualities replaces conventional water resources (surface water and groundwater) and is used as irrigation water for different kinds of crops. Its advantages are mainly embodied in the following two aspects: (1) Since the water source is stable and reliable, it can avoid competition between water for agricultural use and water for other purposes, and thus reduce the pressure on the water supply; (2) Since the nitrogen, phosphorus, potassium, and other nutrients available in reclaimed water can be used as a source of manure, it can promote crop growth, reduce the use of synthetic fertilizers, and thus improve soil properties. However, the system water-soil-plants is an interconnected and interrelated natural ecosystem and is a complicated heterogeneous system. Therefore, reclaimed water irrigation could lead to pollutants. Especially, when heavy metals enter into the soil along with water and then get taken up into crops; after being absorbed in the process of plant growth, the system completes the migration and transformation of pollutants from water to soil to vegetables [3,4]. The content of other elements has no significant difference to that of tap water. Sewage which contains a large quantity of nutrient elements helps to improve soil fertility. Since reclaimed water contains traces of saline elements, long-term irrigation will affect soil permeability. Specifically, an excessive accumulation of saline elements in plant roots changes the soil composition, thus resulting in soil compaction. Certain ions of soil’s saline elements are poisonous and can lead to changes in the physical environment of the soil. The increase of sodium ions reduces the soil porosity, resulting in the decrease of the retention capacity of nutrient elements in soil. The negative and positive effects that reclaimed water has on fertility and pollution have made it remain a hot research topic [5]. However, the complexity of migration and transformation of irrigation water in the soil system also make the effects of reclaimed water on soil fertility difficult to determine.

The advantages of using reclaimed water lie in the fact that the supply is stable, and thus more water can be spared for other uses to relieve the pressure on the water supply [6,7]. However, the water-soil-plant system is a natural, interacted and complicated multi-phase system. There are nutrient substances in the water, such as nitrogen, phosphor, potassium, *etc.* [8,9]. They can work as fertilizer for crops, promote their growth, reduce the use of synthetic fertilizers, and improve the soil arability. Due to economic and technical reasons, some pollutants in sewage are not completely removed [10]. The N and P elements, high quantities of completely saline elements, different kinds of toxic traces and substances (heavy metals, organic pollutants, *etc.*) as well as pathogens may become new sources of pollution. These harmful substances may have negative effects on soil and crops. Will pollutants, especially heavy metals, be brought to the soil by reclaimed water and then be absorbed by crops during their growth, moving from water to soil and then into the vegetables? The heavy metals that have entered the soil and crops will not harm the environment and crops over a limited period of time [11]. Once their accumulation exceeds the volume that soil and crops can support, they will harm the crops and human beings, and cause serious ecological problems. This can explain some people’s fear of vegetables, since they think they contain heavy metals. It is necessary to conduct further research, and therefore this study aims to make it clear whether the vegetables irrigated with reclaimed water during their growth have the potential to harm people’s health.

Both domestic and foreign scholars have conducted researches on this topic [12,13,14]. However, most of the researches focus on physiological and biochemical effects on crops, and only a few of them emphasize the quality, crop fruits, and soil metal contents [15,16]. This study, through drip watering tomatoes with reclaimed water, focuses on the effects of irrigation with reclaimed water on heavy metals in tomatoes and soil revealed by the content and distribution feature in the plants, explores whether reclaimed water, after a secondary treatment, can be used for irrigation and for tomatoes, and analyzes the heavy metal balance in the soil-crop system. The paper provides a reference for the evaluation and safety control of the irrigation type mentioned above.

## 2. Materials and Methods

### 2.1. Experimental Crops

The research was conducted in the “Integrated Demonstration Area of the Agricultural Pollution Control Water Source Area of the South-North Water Diversion Project”, which was jointly built by the Geographical Sciences and Natural Resource Research Institute, the Chinese Academy of Sciences and the Policy and Technology Research Center for the South-North Water Division Project Office of the State Council. The experimental area lies in the southern mountainous area of Maojian District, Shiyan City, Hubei Province, and it is in a subtropical monsoon zone. It has four distinct seasons with long winter and short spring. During the spring, the temperature rises rapidly; the rain is heavier in autumn and there is only a small amount of rainfall and snowfall with moderate temperature during winter. The annual amount of solar radiation is 106.6 Kcal/cm**^2^**, the annual amount of physiological radiation is 50.4 Kcal/cm**^2^**, and the time of sunshine duration is 1925.8 h on average per year. The temperature in a typical year ranges from −14.9 °C to 41 °C with the average of 15.3 °C. The annual accumulated temperature (≥10 °C) is 4936.5 °C. The frost-free period lasts 246 days per year. The amount of average rainfall is 855 mm per year, and it differs greatly from year to year. The amount of rainfall in the flood season (from 1 May, to 20 December) takes up 58%–62% of that of the whole year. During the season the rain is heavy, lasts a short time and can easily wash off the soil on the ground surface. In this research, the experimental soil is the yellow-brown soil, and it weighs between 1.56–1.71 g/cm^3^.

In total, the test site has 36 test plots, and the area of each plot is 2m × 3m. During the test, among the 18 plots of the north section, plots 1–6 and 7–18 were irrigated with clear water, and reclaimed water respectively, while all 18 plots of the south section had always been irrigated with clear water before 2013. Between 2011 and 2014, full reclaimed water treatment, rotation irrigation, and mixed irrigation with reclaimed water and clean water treatment were set in 7–18 plots of the north section and 18 plots of the south section, respectively. The rotation irrigation adopts an alternative irrigation method in order to effectively take advantages of reclaimed water and clean water. During the test, the amount of irrigation water is 450 m^3^/hm^2^, but the irrigation time and frequency are determined according to the actual situation of the growth period.

Tomato plants were selected as the test crop; the reclaimed water irrigation test had been carried out in this greenhouse for two years. Tomato seedlings growing well and uniformly were selected for the test; each of them had three leaves and one core and was 20 cm high. The test ended on 20 October and lasted for three months. Such tomatoes were subject to 40 cm of row spacing, 60 cm of plant spacing and 30,000 plants/hm^2^ of planting density. Sandy clay loam was selected for the test and the soil dry bulk density was 1.4 g/cm^3^. A drop irrigation mode was adopted and a drop irrigation tube was set for each row of crops, of which the head flow was 2 L/h and the irrigation amount was controlled by water meters. The reclaimed water used during the test was the secondary effluent from Hubei Shiyan Sewage Treatment Plant and the clean water was groundwater from the test station.

Table 1 shows the contents of seven heavy metals present within the irrigation water. It can be seen from Table 1 that, besides Hg, the contents of other six heavy metals in the reclaimed water are higher than those in the groundwater from the test station. Among others, the contents of As, Cd, and Cr in the reclaimed water are about twice as high as those in the groundwater, 1.5 times for Pb, and one order of magnitude for Cu and Zn. However, the contents of heavy metals in reclaimed water and groundwater are far lower than the upper limits prescribed in the Agricultural Irrigation Water Quality Standard (GB5084-92).

In the test station, the reclaimed water quality is stable and the water is only brought to the spot at the time of the experiment.

### 2.2. Monitoring Items and Methods

The samples of tomatoes and soil were collected twice, in August 2011 and 2014 respectively, when the plants ripened. The soil samples were taken from layers of depths 0–30, 30–60, and 60–90 cm in each small cell. Test indexes include TN, NO3–N, NH_4_^+^–N, organic nitrogen, available phosphorus, available potassium, Ca**^2+^**, Mg**^2+^**, K**^+^**, Na**^+^**, SO2^−4^, CO_2_^−3^, HCO^3^^−^, pH-value, EC, CEC, As, Cd, Cr, Cu, Hg, Pb and Zn. The sampling time for the plants was August 2014, when the tomatoes ripened, and the samples were the fruits. The test indexes are as follows: seeds and fruit dry weight, TN, NO_-3_-N, NH_+4_–N, TP, As, Cd, Cr, Cu, Hg, Pb, and Zn contents of them.

### 2.3. Instruments and Reagents

**Instruments:** An inductively coupled plasma emission spectrograph (ICP-AES), PHs-10A model digital display ion meter, analytical balance, electric dry oven, bath water kettle, electric vibrating machine, *etc.*

**Reagents:** Nitric acid (guarantee reagent), per chloric acid (which is analytically pure), hydrofluoric acid (analytically pure), hydrogen peroxide, de-ionized water, 1.6000 mol·L^−1^ potassiumdichromate standard solution, 0.4 mol·L^−1^ of ferrous sulfate solution, phenanthro line indicator, paraffin vegetable oil, and concentrated sulfuric acid.

### 2.4. Sample Determination Method

To determine the heavy metal contents in soil and tomatoes: pick the tomatoes twice weekly during the full productive period. The measurement is repeated at each production process and the average taken for the final output data. Pick 10 ripe tomatoes from each small cell; determine the heavy metal contents of the tomatoes. Use the mass spectrometer MDS26 microwave digestion instrument and the inductively coupled plasma ELAN5000 model to determine the heavy metals in the tomatoes [17,18]. Determination method: cut tomatoes into pieces, put 1200 g into the PTFE digestion pot, add 1ml of concentrated HNO_3_ and H_2_O_2_ to each, use the expander for expansion of the packed piston seal and put on the cover of the inner tank. Dry the outer wall of the inner pot. Put the inner pot in the outer pot which is equipped with pads inside. Put the gland on the pot shelf alongside two screws, and start the microwave digestion process. Remove the pot after sufficient cooling. The solution is then quantitatively transferred to a 10 mL tube. Add 20 mg/mL standard internal mixture and set to 10 mL volume. At the operating parameters of ICP2MS, determine the content of As, Pb, the mass concentration of Cd, Hg and other elements in the digested sample solution.

Determine Pb, and Cd in soil by graphite furnace atomic absorption spectrometry and heavy metals such as Cu and Zn, as well as the other contents of the soil by flame atom absorption spectrometry.

### 2.5. Experimental Data Analysis

Analyze all the data with the statistical analysis software SPSS12.

## 3. Results Analysis and Discussion

### 3.1. Heavy Metal Content of Reclaimed Water

In Table 1, there are seven heavy metals in the reclaimed water. As can be seen from the Table, the Hg and Pb contents are similar to those in normal water, while the other heavy metals are higher than those in the normal test water of the experimental area. Among these, the As, Cd, and Cr contents are about twice that of normal water. Cu and Zn are respectively 13 and 15 times higher than that of the normal water. However, the heavy metal contents are much less than the upper limit of the agricultural irrigation water standard (GB5084-08).

### 3.2. Effects of Reclaimed Water of Different Periods on the Content of Heavy Metals in the Soil

Table 2 shows the distribution of seven heavy metals in soil with different reclaimed water irrigation periods. As can be seen from the table, the content of heavy metals decreases with the increase of the depth of the soil, irrespective of the background values (test values in August 2013), the value of the content after the 12 month reclaimed water irrigation (test values in August. 2014 with reclaimed water irrigation), 18 month reclaimed water irrigation (test values in August 2011 with reclaimed water irrigation) or that of the 24 month irrigation reclaimed water irrigation (test values in August 2011 with reclaimed water irrigation). It proves that the reclaimed water irrigation has no obvious effect on heavy metals distribution in the soil layers as long as the irrigation lasts no more than 24 months [19,20]. Compared with the soil environmental quality standard (GB15618-2008), even with a large heavy metal content of the surface layer(0–30 cm)of the soil, the heavy metal contents are much less than the upper limit of the target value of the soil environmental quality standard prescribed level, where the contents are the highest. It indicates that reclaimed water irrigation in the short term (≤24 months) will not cause heavy metal accumulation pollution. This result is consistent with the results of María *et al.* and Adrien *et al.* [21,22].

As is shown in Table 2, by comparing heavy metal contents in 2011 and 2014 with reclaimed water irrigation to the background values with multiple comparisons (LSD method) analysis, it can be observed that the As, Cr, Cu, Hg, Zn contents in the 0–30 cm soil layer showed some differences, but no significant differences at other depths for As, Cr, Cu, Hg, Zn and other heavy metals.

The cause of the difference above is probably the change of sampling locations in various years. Due to the spatial variation of the soil properties, the soil structure, infiltration capacity, clay particle content and organic matter content are not the same in different sampling locations, which results in the heavy metal content fluctuation and differences in different years. Overall, the seven heavy metals do not accumulate in any of the layers in spite of the extended time of the use of reclaimed water in irrigation. In addition, other factors such as differences in irrigation and rainfall infiltration space, atmospheric dust, heavy metal and other crops uptake, *etc.* also have some impaction the content of heavy metals in the soil to some degree.

### 3.3. Effects of Different Volumes of Reclaimed Water Irrigation on the Content of Heavy Metals in Soil

According to the circumstances of the experimental area, during 2011–2014, different water resources were used to irrigate in different test plots accordingly. The plots are irrigated respectively with fully-cleaned water, clean and recycled water alternatively, and underground water [23,24]. The latter two kinds of water quality are called half-reclaimed water and fully-reclaimed water. The heavy metal contents are shown in Table 3.

As can be seen from Table 3, by comparing the values under various circumstances, it can be seen that there is no significant difference in the contents of most heavy metals. For the heavy metal impact on the environment, the reclaimed water can substitute clear water as the source of irrigation water. There is no significant difference in the contents of heavy metals between different volumes of reclaimed water except for Cd and Zn in the soil of 30–60 cm.The difference might be caused by soil spatial variability of the soil sampling locations. This indicates that irrigation with reclaimed water does not cause accumulation of heavy metals in the tomatoes during their growth.

### 3.4. Distribution Characteristics of Heavy Metals in Tomatoes under Reclaimed Water Irrigation

The metals Cr, Cd, Pb, Hg, As, Cu, and Zn metals, which have a major effect on the crop growth and human body food chain, were selected for analysis. The results are shown in Table 4where it can be seen that the heavy metal content distribution has the following characteristics: The distribution characteristics of heavy metals in tomato fruits change with the quantity of reclaimed water used for irrigation [25,26]. The Cr, Cd, Pb, Hg, As, and Cu contents increase along with the increase of the volume of reclaimed water in irrigation. However, by variance analysis, the variation of Cr content is found to reach a significant level of 90% (F = 6.481, sig = 0.081< 0.1), but for Cd, Pb, Hg, As, Cu, and Zn, they do not reach this significant level. The amount of Zn is so small that it could not be detected in the experiment. Although several kinds of ions show an increasing trend in the tomato fruits, they are far below the index in the national standard, which indicates they are safe for people’s health [27,28]. The analysis and comparison above indicate that irrigation with reclaimed water is securer than with sewage. In addition, compared with irrigation with clear water, reclaimed water for irrigation does not cause a significant increase of heavy metal content in tomato fruits. This further indicates that short term reclaimed water irrigation has a very small effect on heavy metal content in the crops.

### 3.5. The Balance Analysis of Content of Heavy Metals in Soil and Tomatoes under Reclaimed Water Irrigation

During their growth in 2011–2014, according to the heavy metals that enter the soil when irrigation is carried out with reclaimed water and those taken away by the above-ground part of the tomato, this paper analyzes the balance of heavy metals in the soil and tomatoes. As is shown in Table 5, for the seven kinds of heavy metals we studied, the amount that is taken away by the above-ground part of the tomatoes is higher than that brought in by reclaimed water. For those irrigated with full-recycled water, the amount of As, Cd, and Hg that was taken away is respectively 11, 13, and 23 times higher than that brought in. For Pb, it is around 31 times higher. Also, for Cr, Cu, and Zn, it is respectively 2, 2.5, and 2 times higher respectively. On analysis of the balance of the heavy metals in soil and crops, it is not difficult to observe that the water helps to discharge heavy metals. The same goes with clear water and mixture irrigation [29,30]. This may affect the heavy metals balance in the soil. However, as we can see from Table 3, the soil heavy metal content does not show significant changes (increase or decrease) before and after the tomatoes growth season. So except for the effect of water quality, the difference in taken-away and brought-in values for the soil heavy metal contents may have a lot to do with the atmosphere, fertilization, and other factors.

The proportion of heavy metals taken away and brought in in the depth of 0–90cm is shown in Table 6. As for those taken away, As is the highest with 0.68%–0.72% while Cr is the lowest, 0.061%–0.070%. Zn has the highest brought-in proportion of 0.02%–0.30%, and Pb accounts for the lowest brought-in proportion, 0.0051%–0.0071%. This shows that both the brought-in and taken-away heavy metal contents account for very small proportions of the total heavy metal contents in the soil depth of 0–90 cm. In conclusion, the brought-in with reclaimed water irrigation and taken away by the above ground part of the crop have little effect on the heavy metal contents balance in the soil.

## 4. Conclusions

(1) Different volumes of reclaimed water have no significant effect on the heavy metal volumes in the soil. For the seven kinds of heavy metals, that taken-away by the crop harvest is always higher than that brought-in when irrigated with reclaimed water. Among all the heavy metals, the taken-away volumes are respectively 11, 13, and 23 times higher than the brought-in for metals such as As, Cd, and Hg. For Pb, the taken-away value is about 31 times the amount brought-in, but both the brought-in and taken-away values account for very small proportions of total heavy metal contents of the soil at a depth of 0–90 cm. This shows that the reclaimed water irrigation has little effect on the heavy metal pollution in the soil.

(2) The field experiment of using reclaimed water to irrigate tomatoes shows that heavy metals in the soil increase, but there is no significant difference. The heavy metal volumes in soil and crops are far below the national soil environmental quality standard and food hygiene permission value standards. Thus, reclaimed water irrigation would not cause accumulation of heavy metal pollution to the soil environment and the crops.

(3) Reclaimed water irrigation is not the only decisive factor for the change in the heavy metals volume in soil and crops; it is also affected by fertilization, soil self-purification capacity, soil and crop types *etc.* The effects of reclaimed water irrigation on crop nutrition, the relationship between reclaimed water quality and the crop yields, crop quality and morphologic change, and the safe contents of organic and the main harmful substances in the reclaimed water that the soil-plant system can support, are all topics that are worthy of further research.

## Figures and Tables

**Table 1:** Heavy metal content of the reclaimed water.

Water Quality	*As* /mg·L^−1^	Cd μg·L^−1^	Cr /mg·L^−1^	Cu /mg·L^−1^	Hg μg·L^−1^	Pb /mg·L^−1^	Zn /mg·L^−1^
Raw wastewater	0.0047	0.060	0.0311	0.0302	0.034	0.0021	0.2102
Underground water	0.0018	0.031	0.0132	0.0017	0.029	0.0010	0.0120
Reclaimed water	0.0039	0.055	0.0240	0.0227	0.022	0.0015	0.1850
Agricultural irrigation water quality standard	0.05	5	0.1	1	1	0.1	2

**Table 2:** Heavy metal content in the soil for different reclaimed water for irrigation periods and background values.

Index	Depth/cm	Background Values	Irrigation Periods			Soil Quality Standard GB15618-2008		
			12 Months	18 Months	24 Months	First Grade	Second Grade	Third Grade
As mg·kg^−1^	0~30	8.4a	9.8b	7.7a	9.5b	≤ 15 ≤ 25 ≤ 40		
	30~60	8.1ab	9.0a	7.1b	8.4a			
	60~90	7.5	8.1	6.6	7.5			
Cd μg·kg^−1^	0~30	130a	132a	133a	120a	≤ 200 ≤ 1000		
	30~60	105a	121a	105a	107a			
	60~90	94	104	88	105			
Cr mg·kg^−1^	0~30	73a	74a	64b	70a	≤ 90 ≤ 250 ≤ 300		
	30~60	71a	70a	66a	67a			
	60~90	71	65	63	68			
Cu mg·kg^−1^	0~30	24a	22b	22b	21b	≤ 35 ≤ 100 ≤ 400		
	30~60	21a	21a	19a	20a			
	60~90	19	20	17	18			
Hg μg·kg^−1^	0~30	44a	48ac	57b	52c	≤ 150 ≤ 1000 ≤ 1500		
	30~60	21a	23a	20a	18a			
	60~90	12	15	15	16			
Zn mg·kg^−1^	0~30	70a	60b	64ab	54c	≤ 100 ≤ 300 ≤ 500		
	30~60	64a	60a	62a	51b			
	60~90	61	51	58	48			

Note: At the level of *p* = 0.05, the same letters stand for no significant difference, different letters stand for the opposite.

**Table 3:** Heavy metal contents in the soils irrigated with the different ratios of clean and reclaimed water.

Index	Depth/cm	Local Values	Full-Clear Water	Half-Reclaimed Water	Two-Thirds-Reclaimed Water	Underground Water
As /mg·kg^−1^	0~30	8.4	9.0a	9.3a	9.6a	9.8a
	30~60	8.1	8.0a	8.7ac	9.3ac	10.0cd
	60~90	7.5	8.0a	8.1a	8.1a	8.2a
Cd /μg·kg^−1^	0~30	130	105a	121a	124a	127a
	30~60	105	96a	132ab	121ab	110c
	60~90	94	94a	104a	107a	90a
Cr /mg·kg^−1^	0~30	73	60a	69a	71a	75a
	30~60	71	63a	67a	70a	76a
	60~90	71	58a	58a	60a	65a
Cu /mg·kg^−1^	0~30	24	17a	21a	21a	22a
	30~60	21	18a	20a	21a	23a
	60~90	19	17a	18a	19a	20a
Hg /μg·kg^−1^	0~30	44	51a	50a	50a	51a
	30~60	21	34a	22a	23a	25a
	60~90	12	18a	15a	17a	19a
Pb /mg·kg^−1^	0~30	70	14a	18a	20a	22a
	30~60	64	17a	18a	19a	21a
	60~90	61	14a	15a	17a	19a
Zn /mg·kg^−1^	0~30	8.4	46a	56a	58a	60a
	30~60	8.1	43ab	52b	57a	61bc
	60~90	7.5	46a	48a	49a	50a

Note: For the level where *p* = 0.05, the same letter means that there is no significant difference, and different letters mean there are apparent differences.

**Table 4:** Tomato fruits heavy metal contents with diverse irrigation water quality mg/kg.

Water Quality	As	Cd	Cr	Hg	Pb	Cu	Zn
Underground water	0.0011	0.018	0.049	0.0028	0.055	0.014	not detected
Half reclaimed water	0.0014	0.029	0.061	0.0031	0.068	0.015	not detected
Underground water	0.0018	0.040	0.082	0.0040	0.075	0.0021	not detected
National standard	0.5	0.05	0.5	0.01	0.2	0.5	not detected

**Table 5:** The amount of heavy metals brought in by the reclaimed water and that taken away when the fruits are harvested during their growth.

Factor	Treatment	As	Cd	Cr	Cu	Hg	Pb	Zn
Heavy metal taken away by the above ground part of the tomato	Underground water	71.2514	0.8979	48.8856	139.9315	0.5721	42.0076	310.4012
	Half-recycled water	45.2678	0.8195	47.4478	84.8757	0.6394	47.8875	289.1785
	Underground water	59.2521	0.8994	58.7648	91.5238	0.6011	49.6679	300.7734
Heavy metal brought-in with the recycled water for irrigation	Underground water	2.7862	0.0501	17.5635	3.6745	0.0512	1.1084	17. 1067
	1/2 reclaimed water	3.9120	0.0549	24.8677	17.1052	0.0417	1.5123	130.9985
	Underground water	5.4268	0.0701	30.9963	28.8649	0.0426	1.6016	250.1007

**Table 6:** The proportion of heavy metals brought in and taken away in the soil during the growth of tomatoes irrigated with reclaimed water.

Factor	Treatment	As	Cd	Cr	Cu	Hg	Pb	Zn
Heavy metal taken-away proportions accounting for the half-recycled water total volume in soil	Underground water	0.7164	0.6928	0.0609	0.5872	0.6298	0.1812	0.4276
	Half-reclaimed water	0.5082	0.6102	0.0524	0.3863	0.6110	0.2011	0.4189
	Full-reclaimed water	0.6826	0.6927	0.0699	0.3547	0.5987	0.2103	0.3978
Heavy metal brought-in proportions accounting for total volume in soil	Underground water	0.02177	0.0405	0.0215	0.0103	0.2198	0.0051	0.0208
	Half-reclaimed water	0.04135	0.0411	0.0278	0.0673	0.1257	0.0060	0.1967
	Full-reclaimed water	0.05017	0.0521	0.0398	0.1257	0.0987	0.0071	0.3057

## References

[1] Yi L., Jiao W., Chen X., Chen W. (2011). An overview of reclaimed water reuse in China. J. Environ. Sci..

[2] Badruzzaman M., Joan A., Oppenheimer J.G. (2013). Impact of environmental conditions on the suitability of micro constituents as markers for determining nutrient loading from reclaimed water. Water Res..

[3] Kayser G.L., Moriarty P., Fonseca C., Bartram J. (2013). Domestic water service delivery indicators and frameworks for monitoring, evaluation, policy and planning: A Review. Int. J. Environ. Res. Public Health.

[4] Rivera-Aguilar V., Godínez-Alvarez H., Moreno-Torres R., Rodríguez-Zaragoza S. (2009). Soil physico-chemical properties affecting the distribution of biological soil crusts along an environmental transect at Zapotitlándrylands, Mexico. J. Arid. Environ..

[5] Becerra-Castro C., Lopes A.R., Vaz-Moreira I., Silva E.F., Manaia C.M., Nunes C.O. (2015). Wastewater reuse in irrigation: A microbiological perspective on implications in soil fertility and human and environmental health. Environ. Int..

[6] Tryland I., Eregno F.E., Braathen H., Khalaf G., Sjølander I., Fossum M. (2015). On-line monitoring of *Escherichia coli* in raw water at oset drinking water treatment plant, Oslo(Norway). Int. J. Environ. Res. Public Health.

[7] Pedrero F., Maestre-Valero J.F., Mounzer O., Alarcón J.J., Nicolás E. (2014). Physiological and agronomic mandarin trees performance under saline reclaimed water combined with regulated deficit irrigation. Agric. Water Manag..

[8] Chen M., Chen J., Sun F. (2010). Estimating nutrient releases from agriculture in China: An extended substance flow analysis framework and a modeling too L. Sci. Total. Envir..

[9] Johannessen G.S., Wennberg A.C., Nesheim I., Tryland I. (2015). Diverse land use and the impact on (irrigation) water quality and need for measures—A case study of a Norwegian river. Int. J. Environ. Res. Public Health.

[10] Liu R., Liu J., Zhang Z., Borthwick A., Zhang K. (2015). Accidental water pollution risk analysis of mine tailings ponds in Guanting reservoir watershed, Zhangjiakou city, China. Int. J. Environ. Res. Public Health.

[11] Islam M.N., Jung H.-Y., Park J.-H. (2015). Subcritical water treatment of explosive and heavy metals co-contaminated soil: Removal of the explosive, and immobilization and risk assessment of heavy metals. J. Environ. Manag..

[12] Mutamim N.S.A., Noor Z.Z., Hassan M.A.A., Olsson G. (2012). Application of membrane bioreactor technology in treating high strength industrial wastewater: A performance review. Desalination.

[13] Lu S., Liang P., Xiao B. (2015). Study on method of domestic wastewater treatment through new-type multi-layer artificial wetland. Int. J. Hydrogen Energ..

[14] Yang W., Song J., Yoshiro H., Jie T. (2015). Exploration and assessment of optimal policy combination for total water pollution control with a dynamic simulation model. J. Clean. Prod..

[15] Koupaie E.H., Eskicioglu C. (2015). Health risk assessment of heavy metals through the consumption of food crops fertilized by biosolids: A probabilistic-based analysis. J. Hazard Mater..

[16] Von Hoffen L.P., Säumel I. (2014). Orchards for edible cities: Cadmium and lead content in nuts, berries, pome and stone fruits harvested within the inner city neighbourhoods in Berlin, Germany. Ecotoxicol. Environ. Saf..

[17] Yang J., Min Q. (2015). A passive and active microwave-vector radiative transfer (PAM-VRT) model. J. Quant. Spectrosc. Radiat..

[18] De Bonis M.V., Caccavale P., Ruocco G. (2015). Convective control to microwave exposure of moist substrates. Part I: Model methodology. Int. J. Heat Mass. Transfer..

[19] Guan Q., Wang L., Pan B., Guan W., Sun X., Cai A. (2016). Distribution features and controls of heavy metals in surface sediments from the riverbed of the Ningxia-Inner Mongolian reaches, Yellow River, China. Chemosphere.

[20] Dragović R., Gajić B., Dragović S. (2014). Assessment of the impact of geographical factors on the spatial distribution of heavy metals in soils around the steel production facility in Smederevo (Serbia). J. Clean Prod..

[21] Salazar M.J., Rodriguez J.H., Nieto G.L., Pignata M.L. (2012). Effects of heavy metal concentrations (Cd, Zn and Pb) in agricultural soils near different emission sources on quality, accumulation and food safety in soybean [Glycine max (L.) Merrill]. J. Hazard. Mater..

[22] Frantz A., Pottier M.A., Karimi B., Corbel H. (2012). Contrasting levels of heavy metals in the feathers of urban pigeons from close habitats suggest limited movements at a restricted scale. Environ. Pollut..

[23] Muhid P., Davis T.W., Bunn S.E., Burford M.A. (2013). Effects of inorganic nutrients in recycled water on freshwater phytoplankton biomass and composition. Water Res..

[24] Chen H., Xia Q.-K., Ingrin J., Jia Z.B., Feng M. (2015). Changing recycled oceanic components in the mantle source of the Shuangliao Cenozoic basalts, NE China: New constraints from water content. Tectonophysics.

[25] Gołdyn B., Chudzińska M., Barałkiewicz D., Celewicz-Gołdyn S. (2015). Heavy metal contents in the sediments of astatic ponds: Influence of geomorphology, hydroperiod, water chemistry and vegetation. Ecotoxicol. Environ. Saf..

[26] Dziubanek G., Piekut A., Rusin M., Baranowska R., Hajok I. (2015). Contamination of food crops grown on soils with elevated heavy metals content. Ecotoxicol. Environ. Saf..

[27] Fierens T., Cornelis C., Standaert A., Sioen I., De Henauw S., Van Holderbeke M. (2014). Modelling the environmental transfer of phthalates and polychlorinated dibenzo-p-dioxins and dibenzofuransin to agricultural products: The EN-forc model. Environ. Res..

[28] Pinto C.E.M., Farias D.F. (2015). Food safety assessment of an antifungal protein from Moringaoleifera seeds in an agricultural biotechnology perspective. Food. Chem. Toxicol..

[29] Hu X.F., Jiang Y., Shu Y., Hu X., Liu L., Luo F. (2014). Effects of mining wastewater discharges on heavy metal pollution and soil enzyme activity of the paddy fields. J. Geochem. Explor..

[30] Gu Q., Deng J., Wang K., Lin Y., Li J., Gan M., Ma L., Hong Y. (2014). Identification and Assessment of Potential Water Quality Impact Factors for Drinking-Water Reservoirs. Int. J. Environ. Res. Public. Health.

